# Supplementary material for: A Critical View of the Application of the APEX Software (Aqueous Photochemistry of Environmentally-Occurring Xenobiotics) to Predict Photoreaction Kinetics in Surface Freshwaters
Source: Molecules. 2019 Dec 18;25(1):9. doi: 10.3390/molecules25010009 (PMC7017383; doi:10.3390/molecules25010009)
Supplement: Supplementary file 1 [file molecules-25-00009-s001.zip › 3.2.4_gcc-4.4.0.zip!/3.2.4_gcc-4.4.0/3.2.4_gcc-4.4.0/doc/octave/html/interpreter/Arithmetic-Ops.html]

Arithmetic Ops - Untitled


Next: Comparison Ops,
Previous: Calling Functions,
Up: Expressions


---

### 8.3 Arithmetic Operators

The following arithmetic operators are available, and work on scalars
and matrices.

x `+` y: Addition. If both operands are matrices, the number of rows and columns must both agree. If one operand is a scalar, its value is added to all the elements of the other operand. x `.+` y: Element by element addition. This operator is equivalent to `+`. x `-` y: Subtraction. If both operands are matrices, the number of rows and columns of both must agree. x `.-` y: Element by element subtraction. This operator is equivalent to `-`. x `*` y: Matrix multiplication. The number of columns of x must agree with the number of rows of y. x `.*` y: Element by element multiplication. If both operands are matrices, the number of rows and columns must both agree. x `/` y: Right division. This is conceptually equivalent to the expression ``` (inverse (y') * x')' ``` but it is computed without forming the inverse of y'. If the system is not square, or if the coefficient matrix is singular, a minimum norm solution is computed. x `./` y: Element by element right division. x `\` y: Left division. This is conceptually equivalent to the expression ``` inverse (x) * y ``` but it is computed without forming the inverse of x. If the system is not square, or if the coefficient matrix is singular, a minimum norm solution is computed. x `.\` y: Element by element left division. Each element of y is divided by each corresponding element of x. x `^` y x `**` y: Power operator. If x and y are both scalars, this operator returns x raised to the power y. If x is a scalar and y is a square matrix, the result is computed using an eigenvalue expansion. If x is a square matrix, the result is computed by repeated multiplication if y is an integer, and by an eigenvalue expansion if y is not an integer. An error results if both x and y are matrices. The implementation of this operator needs to be improved. x `.^` y x `.**` y: Element by element power operator. If both operands are matrices, the number of rows and columns must both agree. `-`x: Negation. `+`x: Unary plus. This operator has no effect on the operand. x`'`: Complex conjugate transpose. For real arguments, this operator is the same as the transpose operator. For complex arguments, this operator is equivalent to the expression ``` conj (x.') ``` x`.'`: Transpose.

Note that because Octave's element by element operators begin with a
., there is a possible ambiguity for statements like

```
     1./m
```

because the period could be interpreted either as part of the constant
or as part of the operator. To resolve this conflict, Octave treats the
expression as if you had typed

```
     (1) ./ m
```

and not

```
     (1.) / m
```

Although this is inconsistent with the normal behavior of Octave's
lexer, which usually prefers to break the input into tokens by
preferring the longest possible match at any given point, it is more
useful in this case.
